# A Conceptual Approach for Examining Effects of the Adolescent Bone Marrow Milieu on MSC Phenotype

**DOI:** 10.1002/jbm4.10740

**Published:** 2023-05-10

**Authors:** Sanjana Kannikeswaran, Daniel G Whitney, Maureen J Devlin, Ying Li, Michelle S Caird, Andrea I Alford

**Affiliations:** ^1^ Department of Orthopaedic Surgery University of Michigan School of Medicine Ann Arbor MI USA; ^2^ Department of Physical Medicine and Rehabilitation University of Michigan Ann Arbor MI USA; ^3^ Institute for Healthcare Policy and Innovation University of Michigan Ann Arbor MI USA; ^4^ Department of Anthropology University of Michigan Ann Arbor MI USA

**Keywords:** BONE MARROW MICROENVIRONMENT, MESENCHYMAL STROMAL CELLS, PEDIATRIC ORTHOPEDIC SURGERY, SECRETOME

## Abstract

Children with bone fragility often exhibit elevated bone marrow lipid levels, which may affect mesenchymal stem cell (MSC) differentiation potential and ultimately bone strength via cell‐autonomous and/or non‐cell‐autonomous factors. Here, we use standard co‐culture techniques to study biological effects of bone marrow cell‐derived secretome on MSC. Bone marrow was collected during routine orthopedic surgery, and the entire marrow cell preparation, with or without red blood cell (RBC) reduction, was plated at three different densities. Conditioned medium (secretome) was collected after 1, 3, and 7 days. ST2 cells, a murine MSC line, were then cultured in the secretomes. Exposure to the secretomes was associated with reductions of up to 62% in MSC MTT outcomes that depended on the duration of secretome development, as well as marrow cell plating density. Reduced MTT values were not associated with diminished cell number and viability assessed using Trypan Blue exclusion. Expression of pyruvate dehydrogenase kinase 4 was modestly elevated, and β‐actin levels were transiently reduced in ST2 cells exposed to secretome formulations that had elicited maximal reductions in MTT outcomes. The findings from this study can inform the design of future experimental studies to examine contributions of cell‐autonomous and non‐cell‐autonomous factors in the bone marrow to MSC differentiation potential, bone formation, and skeletal growth. © 2023 The Authors. *JBMR Plus* published by Wiley Periodicals LLC on behalf of American Society for Bone and Mineral Research.

## Graphical abstract

Consideration of patient‐derived bone marrow as a whole tissue.

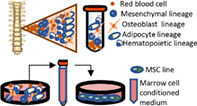

## Introduction

Bone growth during childhood and adolescence includes complex, coordinated processes that are critical for the healthy development of bone mass, structure, and architecture.^(^ ^1^, ^2^ ^)^ Bone marrow tissue, which includes heterogeneous cell lineages, influences bone development.^(^ ^3^, ^4^, ^5^, ^6^ ^)^ For example, marrow adipocytes and osteoblasts arise from a shared mesenchymal stromal cell (MSC) precursor, and when MSC differentiation into adipocytes is enhanced, osteogenic differentiation is contrarily suppressed.^(^ ^7^, ^8^ ^)^ Post‐differentiation functions of adipocytes can also adversely affect bone metabolism and influence MSC differentiation toward adipogenic pathways.^(^ ^9^, ^10^, ^11^ ^)^ Additionally, immune cells arising from hematopoietic stem cells in the bone marrow may influence bone biology through various signaling pathways.^(^ ^3^, ^4^, ^12^, ^13^ ^)^ Megakaryocytes, T cells, and B cells can have differential effects on osteogenesis depending on their activation status and protein expression profiles.^(^ ^3^, ^4^, ^13^ ^)^ Furthermore, hematopoietic progenitors within the bone marrow give rise to osteoclasts, and these cells' role in bone resorption has been well documented.^(^ ^14^ ^)^ Taken together, bone marrow encompasses a complex and dynamic environment with many cell–cell relationships and signaling activities that can biologically influence bone metabolism. However, little is known about the pathways influencing interactions between bone marrow constituents and bone metabolism in children, which is further complicated by limited access to bone marrow for direct probing of these biological interactions at the tissue level.

In children, the few studies to investigate bone marrow have focused on fat alone and relied on analysis of tissue distribution (eg, through in vivo imaging) or composition phenotyping (eg, lipid composition) rather than elucidating biological interactions. For example, studies using magnetic resonance imaging among pediatric groups with impaired bone development found that children with cerebral palsy had elevated bone marrow fat in the mid‐tibia,^(^ ^15^ ^)^ that adolescent girls with anorexia nervosa display accelerated conversion of hematopoietic marrow to fatty marrow,^(^ ^16^ ^)^ and that girls with severe anorexia nervosa had elevated bone marrow fat compared with those with more moderate disease.^(^ ^17^ ^)^ Because abnormal bone marrow lipid composition can alter bone biology and is a potential biomarker for osteoporosis,^(^ ^18^ ^)^ these findings prompted further examination of bone marrow lipid composition among children with various degrees of bone fragility, including patients with adolescent idiopathic scoliosis, using lipidomics.^(^ ^19^, ^20^ ^)^ Though MRI and lipidomics offer insight into pediatric bone marrow fat distribution and composition, they are not positioned to study biological interactions between different bone marrow cell lineages and the subsequent effects on bone development. For medically complex pediatric populations with bone fragility, distinct cell populations within the bone marrow microenvironment may influence bone growth and development. Before examining interactions between individual cell lineages, methodological approaches that treat bone marrow as a whole tissue should first be considered. Tissue‐level investigation is positioned to provide novel insights into mechanisms that better reflect in vivo physiology, providing a broader, more translational view to help generate granular, cell‐specific, and mechanistic hypotheses.

The long‐term goal of our work is to develop methodology to enable future research examining biological interactions between various bone marrow cell lineages and bone development in children. The aim of this study was to determine the effect of patient‐derived bone marrow secretome (conditioned medium) on mesenchymal stem cell number and metabolism. To standardize the cells exposed to individual patient‐derived secretomes, we utilized ST2 cells,^(^ ^21^ ^)^ a well‐characterized and readily available murine MSC line that, like human MSC, possess adipogenic^(^ ^22^ ^)^ and osteogenic potential.^(^ ^23^ ^)^ To establish that biologically active secretomes can be prepared from pediatric bone marrow, we used tissue collected during routine clinical surgery and varied the method of marrow cell harvest, marrow cell plating density, and the duration of secretome generation. ST2 cells were cultured in the secretomes, and cell number, metabolism and gene expression were assessed. Biological activity was defined by comparing outcome measures obtained from ST2 cells cultured in secretome to those cultured in control medium. We propose that this conceptual approach can be used and built upon to study different bone marrow cell lineages and their interactions with each other and the bone marrow milieu (Fig. 10).

## Materials and Methods

### Marrow collection

Eighteen patients were enrolled in the study and their parents provided informed consent. During routine posterior spinal fusion surgery, bone marrow samples from the pedicle and vertebral body of the lumbar and inferior spine of children aged 8 to 18 years with cerebral palsy (CP [*n* = 2 M, 4 F]) and adolescent idiopathic scoliosis (AIS [*n* = 4 M, 8 F]) were collected into 20 mL harvest medium (αMEM containing 2% FBS, 100 IU/mL penicillin, 100 μg/mL streptomycin, and 2 mM glutamax). These procedures were approved by the Institutional Review Board at the University of Michigan, and bone marrow samples were de‐identified.

### Preparation of marrow cell‐derived secretome

**Fig. 1:**
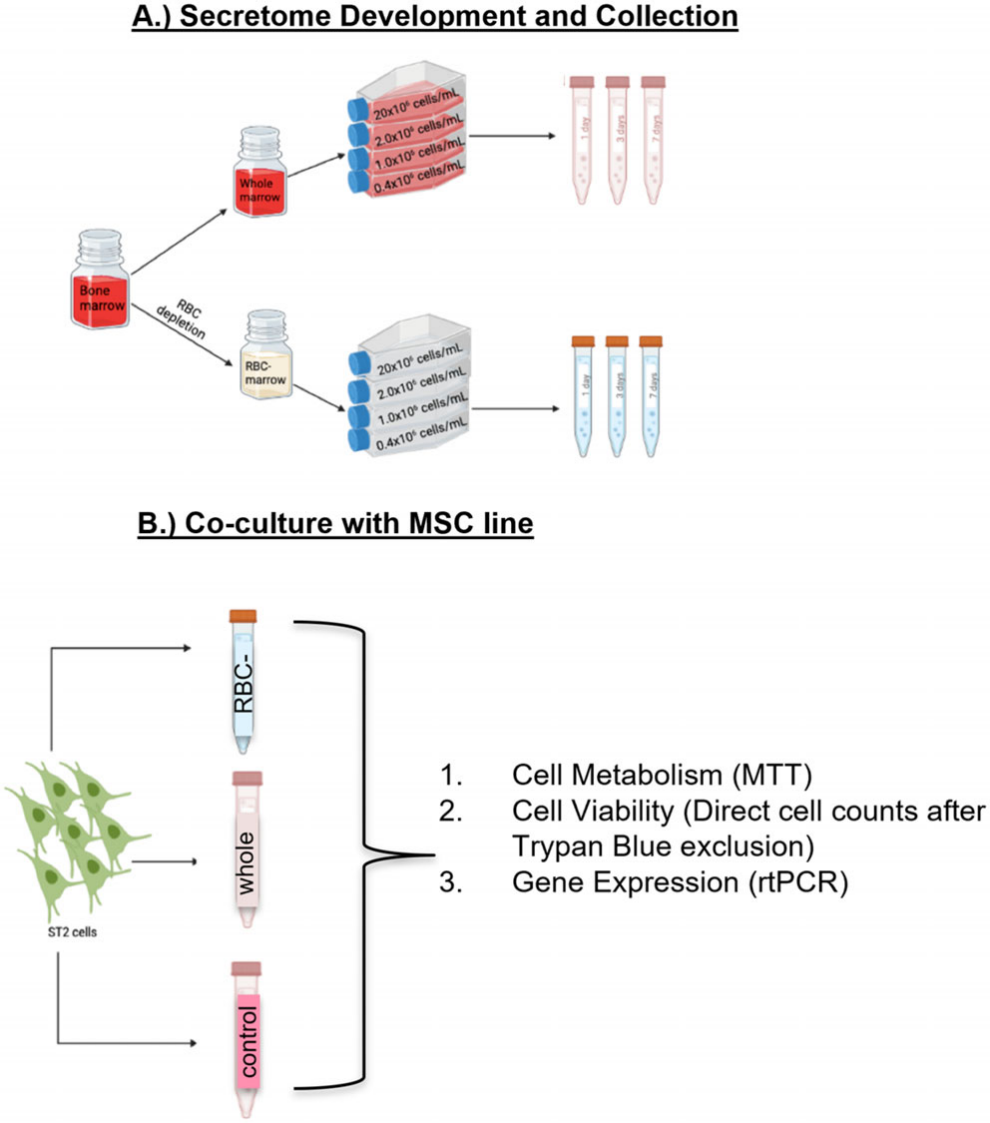
Experimental overview. Secretomes were generated using all cell types constituting bone marrow (*A*; red), or red blood cells were depleted by lysis (*A*; clear). ST2 cells were then cultured in the secretomes (*B*). Effects of the total number of marrow cells present during secretome development, the duration of secretome development, and RBC reduction on ST2 cell metabolism, cell number, and gene expression were evaluated.

The entire process of secretome production (conditioned medium collection) and subsequent co‐culture is outlined in Fig. 1. Immediately after surgery, bone marrow samples were brought up to 40 mL in harvest medium. Single‐cell suspensions were obtained by passing the cells sequentially through a serological pipette, a syringe fitted with a 21G needle, and a 70‐micron cell strainer. Alternatively, after mixing the marrow sample with a serological pipette, it was washed extensively over the cell strainer without using the needle. The resulting single‐cell suspensions were then split in half. One half was subject to red blood cell lysis using 150 mM NH_4_Cl, 10 mM KHCO_3_, and 0.1 mM EDTA in ddH_2_O for 10 minutes at room temperature.^(^ ^24^ ^)^ After washing twice with PBS, an aliquot of this red blood cell (RBC)‐reduced cell preparation was used to determine mononuclear cell density by manually counting using a hemocytometer. Using the cell count data from the RBC‐reduced samples, whole and RBC‐reduced marrow preparations were plated on T75 flasks at 0.4 × 10^6^, 1.0 × 10^6^, 2.0 × 10^6^, and 20.0 × 10^6^ cells per mL in 25 mL MSC growth medium (αMEM containing 10% FBS, 100 IU/mL penicillin, 100 μg/mL streptomycin, and 2 mM glutamax). After 1, 3, and 7 days, 8 mL of conditioned medium was collected, centrifuged for 5 minutes at 500*G* to remove nonadherent cells and debris, divided into 2 mL aliquots, and stored at −80°C. To avoid diluting the secretomes, the volume of medium removed with each collection was not replaced. Secretome aliquots were thawed at 37°C before being utilized immediately in experiments with ST2 cells. Secretomes were not re‐frozen.

### Co‐culture of ST2 cells with patient marrow‐derived secretomes

ST2 cells were thawed, seeded onto T75 flasks in MSC growth medium, and grown to 70% to 80% confluence before passage using trypsin–EDTA. Medium was replaced every 3 days after initial seeding. Experiments were conducted after 2 to 3 passages. For experiments, ST2 cells were resuspended in previously prepared secretomes or in MSC growth medium at 5 × 10^3^ cells per 200 μL and seeded onto 96‐well plates (Fig. 1*B*). Cells were incubated for 48 hours at 37°C before evaluation of cell metabolism using the MTT assay. In additional experiments, ST2 cells (15 × 10^4^) were resuspended in 2 mL secretome or MSC growth medium and seeded onto 6‐well plates. These cultures were maintained for 24 or 72 hours before direct cell counting and preparation of RNA.

### Determination of cell metabolic activity

MTT assays were conducted according to the manufacturer's protocol (Invitrogen, Carlsbad, CA, USA; cat. no. V13154). After 48 hours, medium or secretome was removed and replaced with 100 μL of fresh MSC growth medium made with phenol‐red free αMEM. An amount of 10 μL of 12 mM MTT stock solution was added to each well, and the plate was subsequently incubated for 4 hours at 37°C. All but 25 μL of medium was then removed from the wells, 100 μL 10% SDS solution was added, and incubated overnight at 37°C to dissolve the dark blue formazan crystals. Absorbance values were obtained at 570 and 690 nm using a Synergy HTX plate reader (Agilent, Santa Clara, CA, USA). The results are reported as optical density values (OD570‐OD690) expressed relative to those obtained from cells cultured in MSC growth medium.

### Determination of cell number

After 24 or 72 hours of culture in secretome or growth medium, ST2 cells were treated with trypsin–EDTA, collected, and the total number of live cells were determined by Trypan Blue exclusion using an automated hemocytometer (Countess, Thermo Fisher Scientific, Waltham, MA, USA). After counting, cells were recovered by centrifugation, lysed in Trizol, and stored at −80°C in preparation for RNA extraction.

### RNA extraction and rtPCR

RNA was extracted from cell pellets dissolved in Trizol by adding chloroform and the precipitated using isopropanol. Yield and purity were determined spectrophotometrically (Thermo Fisher Scientific Nanodrop 2000 spectrophotometer). An amount of 200 ng RNA was reverse transcribed using qScript‐cDNA supermix (Quanta Biosciences, Gaithersburg, MD, USA). Expression of PDK4, ppargc1α, and β‐actin were determined with primers designed using RefSeq mRNA sequences and Primer Blast (NCBI). The mitochondrial membrane protein ATP5b served as the internal control. Thermal cycling was performed using a 7500 Fast Real‐Time System (Applied Biosystems, Foster City, CA, USA). Proper amplicon formation was confirmed by agarose gel electrophoresis and by melt‐curve analysis. Relative mRNA expression levels were calculated using the double delta‐Ct method.

### Statistical analysis

All data are presented as mean ± SD, and each point represents data obtained from ST2 cells cultured in secretome prepared as indicated from a single donor. Effects of duration, cell concentration, and RBC‐lysis during secretome generation on MTT outcomes in ST2 cells were determined using repeated measures ANOVA and Tukey post hoc tests (*n* = 6 technical replicates per biologic replicate). Two‐way repeated measures ANOVA was used to determine the effects of time and secretome formulation on ST2 cell number, viability, and gene expression (*n* = 2 to 4 technical replicates per biological replicate). Comparisons between two groups were made using *t* tests. A value of *p* < 0.05 was considered significant. Statistical analysis was conducted using GraphPad (La Jolla, CA, USA) Prism v8.0.0.

### Exploratory analysis

The bone marrow microenvironment is anticipated to be heterogenous across adolescent transitioning. We therefore explored the association between age and MTT outcomes using unadjusted linear regression. Because the needle extrusion step significantly reduced cell yield, this exploratory analysis was only conducted on the 8 samples that were processed without the needle. As such, findings should be interpreted as exploratory and used to inform future study designs. The sample size was too small to examine effects by other patient‐level factors, such as sex, race, or body composition.

## Results

### Optimization of the marrow cell collection method

**Fig. 2:**
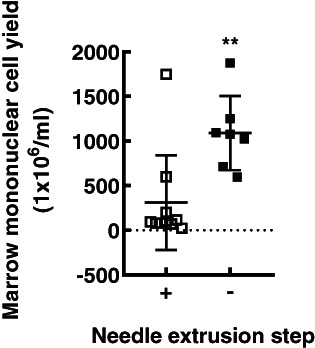
Processing whole bone marrow using needle extrusion reduces total cell yield. Data are mean and SD of total cell counts obtained after generation of a single marrow cell suspension using a 21G needle and a cell filter (*n* = 10, open squares) or a cell filter only (*n* = 7, closed squares) **p < 0.01.

Most surgeries occurred in 2019 (*n* = 16) with the remainder occurring in 2020 (*n* = 1) and 2021 (*n* = 1). The total volume of marrow collected was generally 2.5 to 3 mL, with a small number of surgeries yielding as much as 5 mL of material. Initially, we utilized two different methods for generating single‐cell suspensions from bone marrow. Ten samples (6 [60%] female) were processed using a 21‐gauge needle and a 70‐micron cell strainer, similar to methods used to generate single cell‐suspensions from mouse bone marrow.^(^ ^25^ ^)^ Eight samples (6 [75%] female) were processed using the cell strainer only. Ages were not different (needle method median age 14.5 years, range 8 to 17; cell strainer method age 15.5 years, range 10 to 18). Total cell yields were higher when the needle extrusion step was omitted (1090 ± 415 versus 311 ± 530 × 10^6^ total cells *p* < 0.01; Table 1 and Fig. 2).

**Table 1:** Patient Information, Marrow Cell Harvest Protocol, and Primary Cell Yield

Sample ID	Sex	Age (years)	Diagnosis	Marrow cell preparation method	Cell yield (10^6^ cells)
1	F	11	AIS	Needle	80
2	F	12	AIS	Needle	70
3	M	17	AIS	Needle	22
4	M	17	AIS	Needle	1747
5	F	16	AIS	Needle	104
6	M	16	CP	Needle	115
7	M	16	CP	Needle	598
8	F	16	AIS	Needle	95
9	F	13	AIS	Needle	198
10	F	8	CP	Needle	80
11	F	17	AIS	Cell strainer	1874
12	M	12	AIS	Cell strainer	Not recorded
13	M	17	AIS	Cell strainer	1024
14	F	14	AIS	Cell strainer	1076
15	F	17	CP	Cell strainer	1252
16	F	10	CP	Cell strainer	594
17	F	12	CP	Cell strainer	1095
18	F	18	AIS	Cell strainer	714

Abbreviations: AIS = adolescent idiopathic scoliosis; CP = cerebral palsy.

### Effect of marrow cell dose and duration of development on secretome bioactivity

**Fig. 3:**
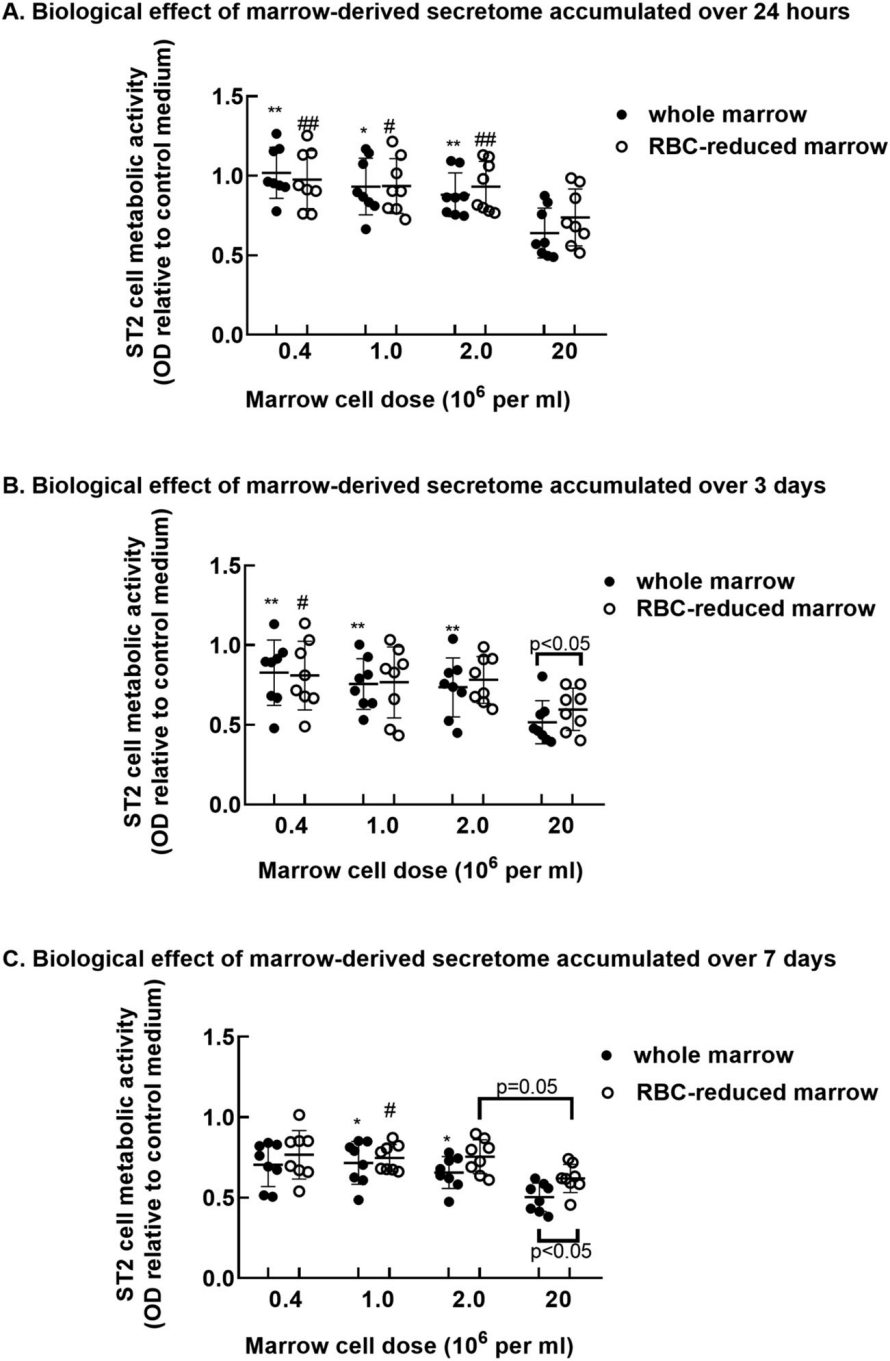
Effect of marrow cell density on biological potency of secretomes generated for 1, 3, and 7 days. Data are mean and SD of MTT outcomes in ST2 cells co‐cultured in secretomes derived from the marrow of 8 donors. Whole and RBC‐reduced bone marrow‐derived secretomes were generated for up to 1 week using varying cell densities. *p < 0.05 and **p < 0.01 versus 20 × 10^6^ whole bone marrow‐derived secretome; #*p* < 0.05 and ##*p* < 0.01 versus 20 × 10^6^ RBC‐reduced bone marrow‐derived secretome by repeated measures ANOVA and Tukey post test.

**Fig. 4:**
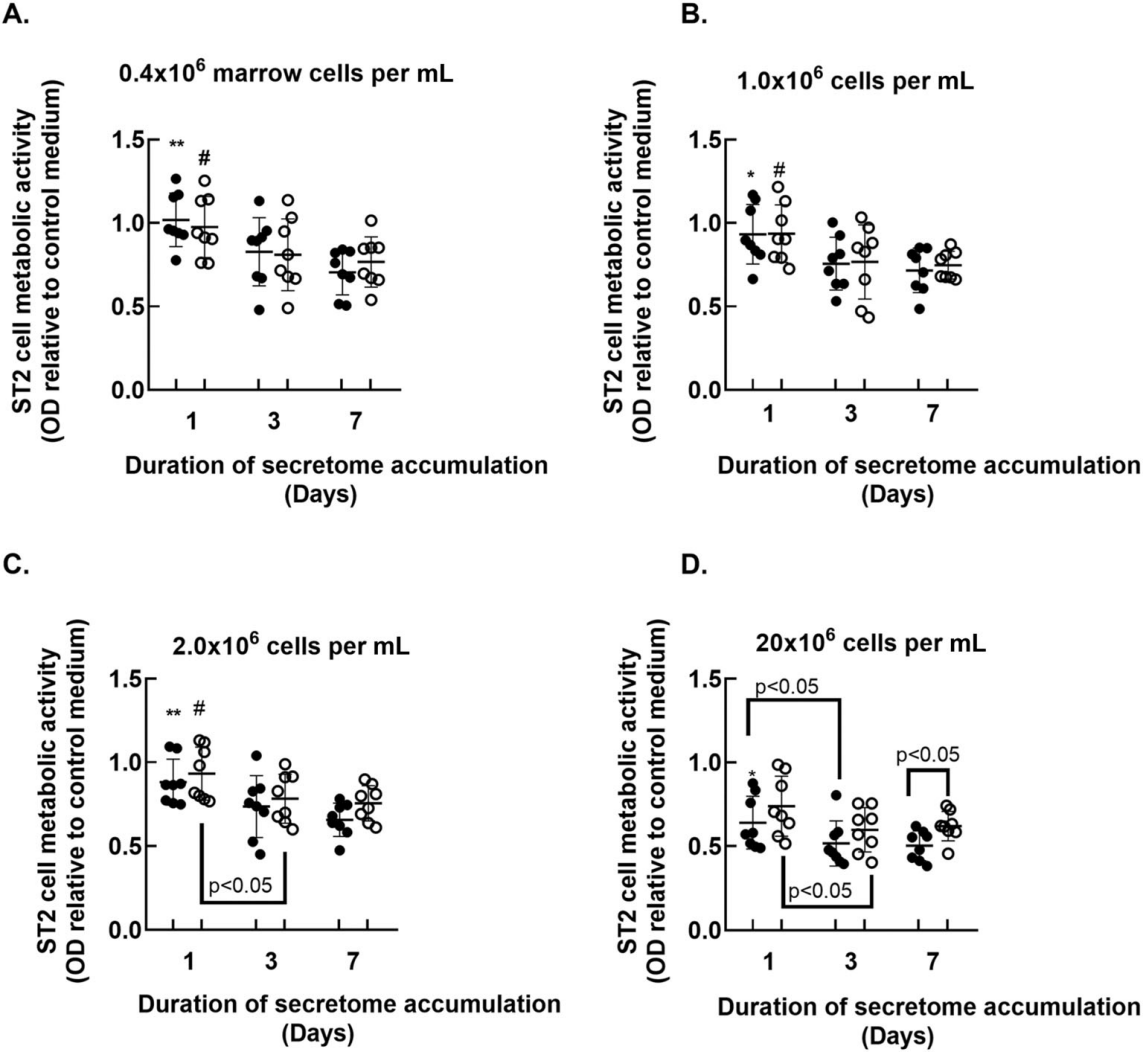
Effect of the duration of secretome development at each marrow cell dose on ST2 cell MTT outcomes. MTT outcomes presented in Fig. 3 (mean and SD) are presented as a function of the duration of secretome development. *p < 0.05 and **p < 0.01 versus whole bone marrow secretome generated for 7 days; #*p* < 0.05 versus RBC‐reduced bone marrow secretome generated for 7 days by repeated measures ANOVA and Tukey post test.

The higher cell yields associated with omitting the needle extrusion step suggested that this methodology captured a more comprehensive sampling of marrow cell types. Thus, secretomes generated using the cell strainer were evaluated further (*n* = 8). ST2 cells were cultured in secretomes or in MSC growth medium as outlined in Fig. 1*B*. MTT assays were conducted 48 hours later. The MTT results in Figs. 3 and 4 are presented relative to data obtained from ST2 cells cultured in MSC growth medium, which is set at 1.0.

Regardless of RBC reduction, ST2 cells co‐cultured in secretomes that had been generated for 1 day using 20 × 10^6^ cells per mL displayed reduced MTT outcomes compared with those cultured in secretomes generated using lower cell densities (Fig. 3*A*). ST2 cells co‐cultured in whole bone marrow‐derived secretomes that had been generated for 3 days using 20 × 10^6^ cells per mL displayed reduced MTT outcomes compared with those cultured in secretomes generated using all other whole marrow cell doses (Fig. 3*B*, solid circles). RBC‐reduced secretomes that had developed for 3 days using the highest cell dose induced reductions in ST2 cell MTT outcomes compared with those generated using the lowest RBC‐reduced cell density formulation (Fig. 3*B*, open circles). ST2 cells co‐cultured in whole bone marrow‐derived secretomes generated for 7 days using 20 × 10^6^ cells per mL displayed reduced MTT outcomes compared with those cultured in whole bone marrow‐derived secretomes generated for 7 days using 1.0 and 2.0 × 10^6^ cells per mL (Fig. 3*C*, solid circles). ST2 cells cultured in RBC‐reduced bone marrow‐derived secretomes generated for 7 days using 20 × 10^6^ cells per mL displayed reduced MTT outcomes compared with those cultured with RBC‐reduced secretomes generated using 1 × 10^6^ cells (Fig. 3*C*, open circles). RBC reduction attenuated secretome‐associated reductions in ST2 cell MTT outcomes for secretomes generated for 3 and 7 days using 20 × 10^6^ cells per mL (Fig. 3*B*, *C*).

To more directly evaluate effects of the duration of secretome accumulation on MTT outcomes in co‐cultured ST2 cells, we plotted values from each cell dose against time (Fig. 4). Whole and RBC‐reduced secretomes generated for 7 days using the three lower cell doses reduced ST2 cell MTT outcomes compared with those generated for 1 day (Fig. 4*A*–*C*). RBC‐reduced secretomes generated for 3 days using 20 × 10^6^ cells elicited greater reductions in MTT values compared with those generated for 1 day (Fig. 4*D*).

Secretomes generated with higher bone marrow cell concentrations may have effectively been created starting with serum concentrations of less than 10%. Thus, reduced serum content relative to secretome may explain reduced MTT outcomes in ST2 cells co‐cultured with secretome generated using high marrow cell densities. To address this possibility, the MTT assay was conducted on ST2 cells cultured in medium containing decreasing serum concentrations. When cells were cultured in 5% FBS, MTT values were 0.97 ± 0.14 relative to cells cultured in standard growth medium with 10% FBS. Thus, serum‐dependent declines in MTT outcomes were minimal compared with those associated with the secretomes.

**Fig. 5:**
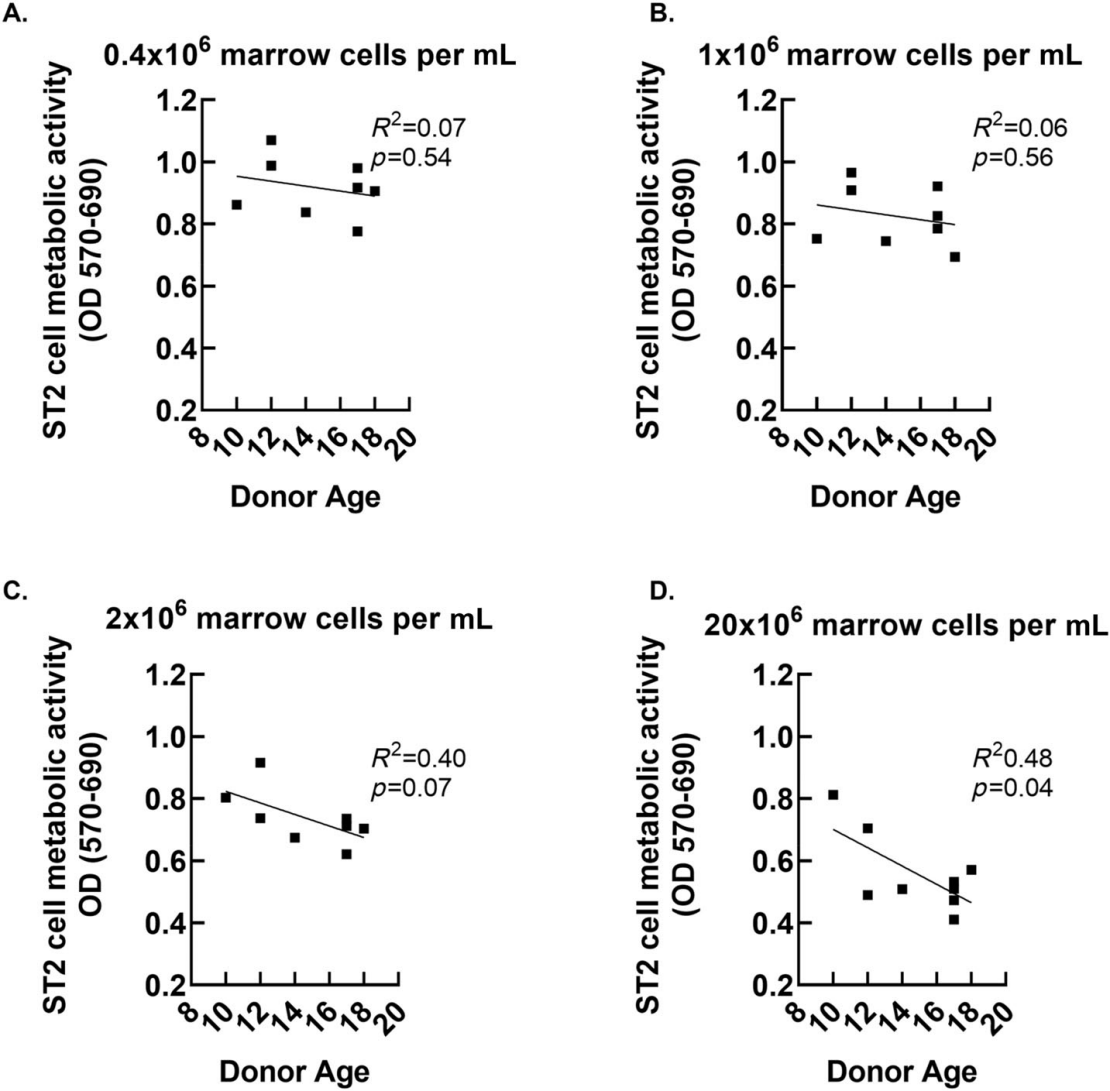
Relationship between donor age and biological activity of whole bone marrow secretomes allowed to develop for 1 day. ST2 cell MTT outcomes are plotted against donor age. The *p* values refer to significance against slope = 0.

**Fig. 6:**
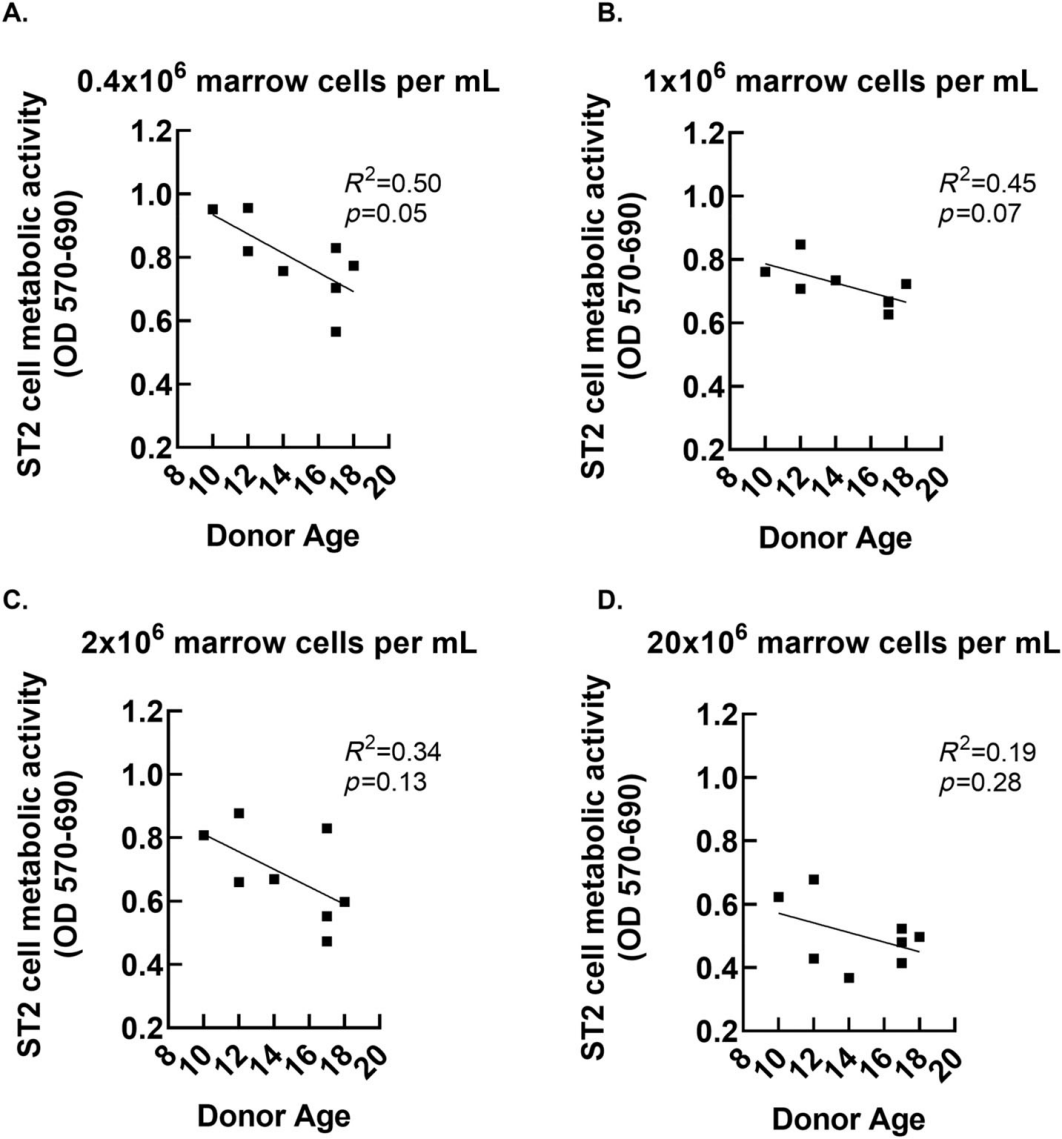
Relationship between donor age and biological activity of whole bone marrow secretomes allowed to develop for 3 days. ST2 cell MTT outcomes are plotted against donor age. The *p* values refer to significance against slope = 0.

**Fig. 7:**
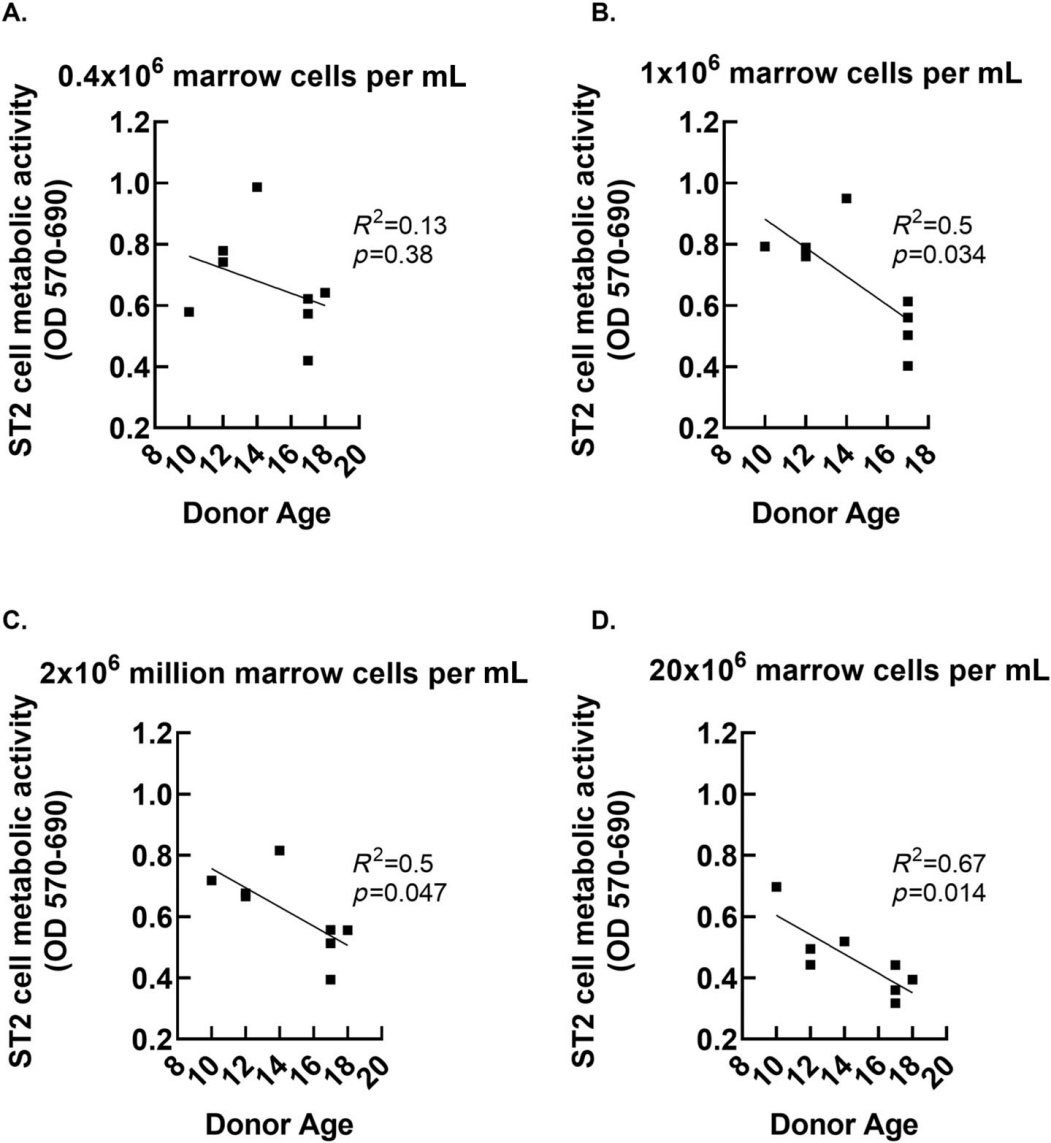
Relationship between donor age and biological activity of whole bone marrow secretomes allowed to develop for 7 days. ST2 cell MTT outcomes are plotted against donor age. The *p* values refer to significance against slope = 0.

Our sample size was too small to test for the effects of sex, age, and other patient level factors on the generation of biologically active secretomes. However, because the cohort of marrow donors represented a broad range of ages relative to puberty, (10–18) we conducted an exploratory comparison of age versus MTT outcomes. The data shown in Figs. 5, 6, 7 suggest a potential negative relationship between donor age and secretome‐associated MTT outcomes. The slopes were significantly different from zero after 1 day of secretome development using the highest cell dose (Fig. 5*D*), after 3 days of secretome development using the lowest cell dose (Fig. 6*A*), and after 7 days of secretome development using the three higher cells doses (Fig. 7*B*–*D*).

MTT values are often reflective of cell number, but they can also indicate differences in cell metabolism. To determine whether reduced cell numbers explain reductions in MTT values, we selected the secretome preparations that had elicited the largest effect on MTT outcomes from 6 patients (Table 2) and conducted another ST2 cell co‐culture experiment in which the cells were counted and then processed for gene expression analysis.

**Table 2:** The Most Biologically Potent Secretome Preparations From 6 Bone Marrow Samples Were Selected for Further Analysis

Sex	Age (years)	Diagnosis	Relative MTT reduction
F	17	AIS	0.57
F	14	AIS	0.43
F	17	CP	0.41
F	10	CP	0.52
M	12	AIS	0.48
F	12	CP	0.55

Abbreviations: AIS = adolescent idiopathic scoliosis; CP = cerebral palsy.

**Fig. 8:**
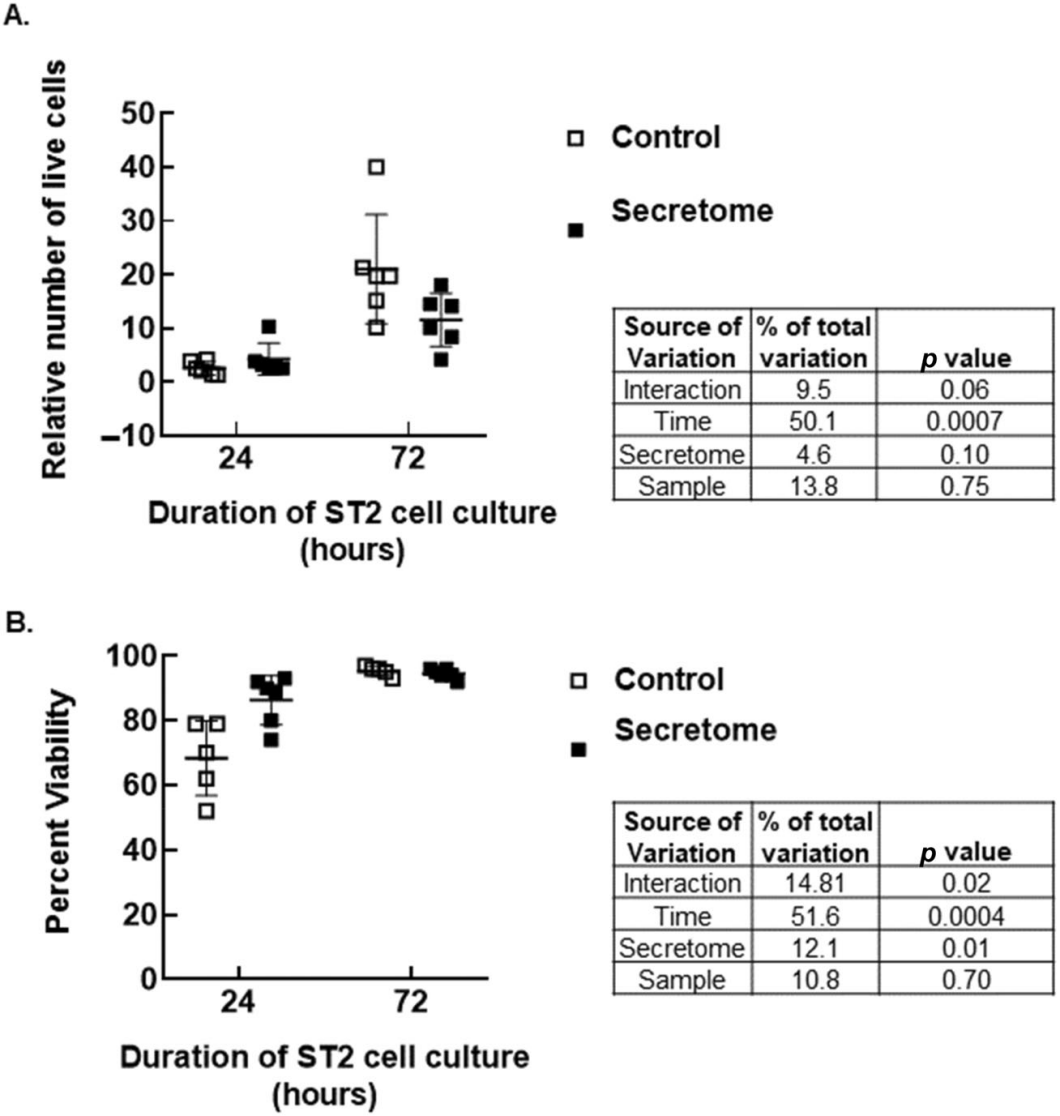
Direct cell counts are not reflective of MTT results. Cell number (*A*) and viability (*B*) increased with time, but they were not impacted by exposure to secretomes previously demonstrated to elicit reductions in MTT outcomes. Data were compared using repeated‐measures two‐way ANOVA.

After 24 or 72 hours of culture, the number of live ST2 cells in secretomes was compared with that in MSC growth medium. Time, but not secretome, impacted the number of cells relative to plating density (Fig. 8*A*). Time also had a significant impact on percent viability (Fig. 8*B*). At the 24‐hour time point, percent viability was higher in secretome, but this difference was transient.

### Secretomes that affected MTT outcomes also affected gene expression

**Fig. 9:**
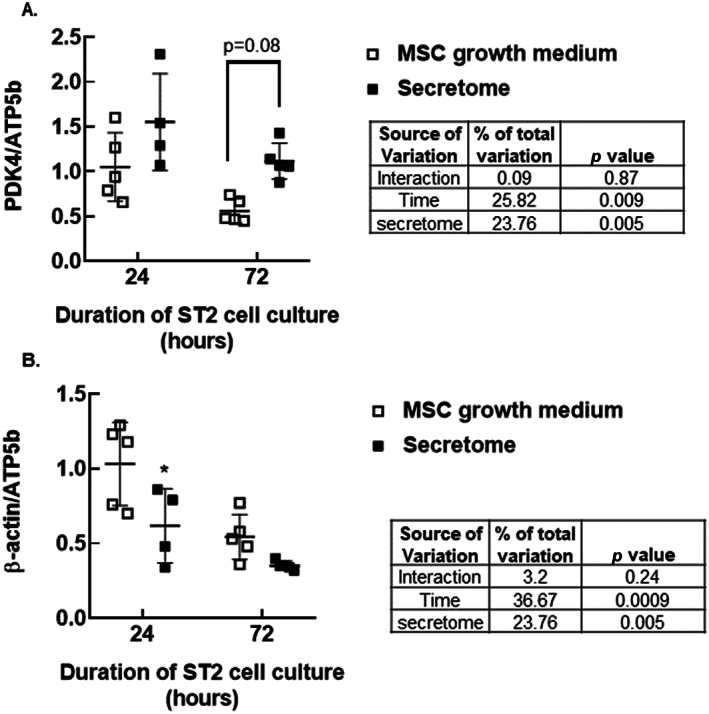
Secretomes that elicited reductions in MTT values also affected gene expression. Data are mean and SD of 4 to 6 biologic replicates per culture condition and time point. *p < 0.05 by two‐way ANOVA and Tukey post tests compared with gene expression values for cells cultured in MSC growth medium for the same duration.

After direct cell counting, cells were processed for RNA extraction and rtPCR. Gene expression values were normalized to the housekeeping gene ATP5b, which did not change with experimental conditions, and expressed relative to data obtained from cells cultured 24 hours in growth medium. Two‐way ANOVA suggested that both time and secretome exposure affected pyruvate dehydrogenase kinase 4 (PDK4) expression. Thus PDK4 decreased between 24 and 72 hours when cells were cultured in MSC growth medium (Fig. 9*A*, open squares) or in secretomes (Fig. 9*A*, closed squares). At 72 hours, cells cultured in secretome displayed a trend toward elevated PDK4 levels compared with those cultured in MSC growth medium (*p* = 0.08 by Tukey post hoc analysis). Two‐way ANOVA suggested that time and secretome exposure also affected β‐actin gene expression (Fig. 9*B*). At 24 hours, β‐actin gene expression was lower in cells cultured in secretome compared with those cultured in MSC growth medium (*p* < 0.05 by Tukey post hoc analysis). We also assessed ppargc1α expression. Even with confirmation of proper amplicon formation via melt curve analysis and gel electrophoresis, ppargc1α levels were very low, making quantification difficult (data not shown). Thus, only secretome‐dependent increases in ppargc1α expression would have been detected.

## Discussion

Perturbations in the bone marrow microenvironment may contribute to skeletal fragility, particularly in growing children. However, interactions of bone marrow and bone metabolism are complex and challenging to study in vivo. Establishing the biological activity of patient bone marrow‐derived secretome in vitro is a necessary step in developing methods to study the biological interactions between various bone marrow cell lineages, MSC differentiation potential, and pediatric bone development. In this study, we sought to determine whether biologically active secretomes can be prepared from pediatric bone marrow and tested the effects of different methods of marrow cell harvest, marrow cell plating density, and duration of secretome generation on cell number and gene expression in ST2 cells exposed to the secretomes in co‐culture. Our results suggest that secretome developed from pediatric bone marrow tissue is biologically active as observed from a secretome‐induced reduction in ST2 cell metabolism and changes in gene expression.

Initially, we generated single‐cell suspensions by sequentially disrupting the marrow sample with a serological pipette, a 21G needle, and a cell strainer to generate the final cell suspension. This protocol resulted in lower than expected yields, so moving forward, we omitted the needle extrusion step. Instead, after mixing with the serological pipette, we washed the marrow tissue sample extensively over the cell strainer, and this resulted in a higher cell yield. Using the needle to generate a single‐cell suspension is likely mechanically disruptive and may cause damage to cells as they are forced through a narrow opening. Because our goal was to generate secretomes from the entire bone marrow cell constituency, we utilized the secretomes generated by cell preparations processed without the needle in subsequent co‐culture experiments.

Secretomes were co‐cultured with ST2 cells to elucidate effects of RBC reduction, marrow cell dose, and duration of secretome development on cell metabolism. Overall, the secretome‐induced decrease in ST2 cell metabolism was dose‐ and time‐dependent, with secretomes that were developed using high cell density or longer durations eliciting significant reductions in ST2 cell metabolism (Figs. 3 and 4). For secretomes generated using the lowest cell dose (0.4 × 10^6^ cells per mL), a time‐dependent increase in the magnitude of MTT reduction was evident (Fig. 4*A*). Secretomes generated using the highest cell concentration (20 × 10^6^ cells per mL) elicited maximum reductions in ST2 cell metabolic activity after 3 days of development (Fig. 4*D*). Thus, the potency of secretomes generated using high cell doses may plateau after a relatively short culture interval. Conversely, using lower cell doses to generate the secretomes may allow accumulation of biologically active components over several days (Fig. 4*A*–*C*).

**Fig. 10:**
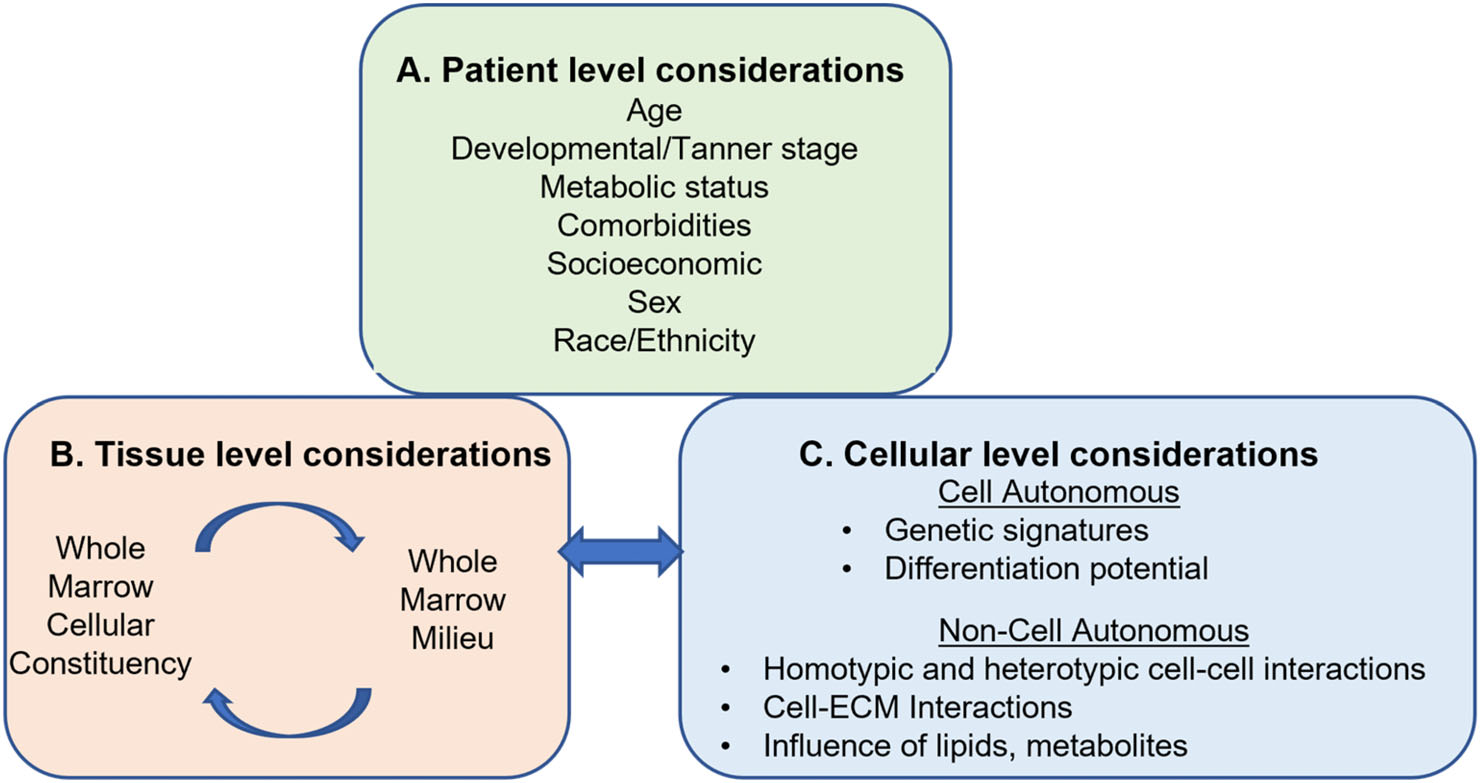
Considerations for design and interpretation of ex vivo analysis of tissues collected from adolescent patients during orthopedic surgery. (*A*) Patient level factors may influence the bone marrow cellular and extracellular milieu and outcomes of ex vivo analyses. (*B*) At the tissue level, bone marrow is a complex mixture of mesenchymal, immunogenic, and erythroid cell types that all influence and respond to the bone marrow milieu and could impact MSC differentiation potential and bone mass accrual. (*C*) Experiments designed to characterize function or transcriptomic profiles of patient‐derived bone marrow at the level of individual cell populations should consider cell‐autonomous factors together with non‐cell‐autonomous and tissue level factors.

The donors represented a broad range of ages relative to puberty, (10–18) so we conducted an exploratory analysis to determine whether age and secretome bioactivity might be related. Figures 5, 6, 7 suggest an overall negative relationship between patient age and MTT outcomes that was evident after 24 hours of secretome development when the highest cell dose was utilized and after 7 days of secretome development for the intermediate and high cell doses. This relationship was observed after 3 days of secretome development with the lowest cell dose tested. For future studies, knowing that biologically active secretomes can be generated using relatively low cell doses allows for the possibility of maximizing information that can be collected from a single specimen. For example, an individual marrow sample could be divided for conducting cellular phenotyping and secretome analyses in parallel (Fig. 10).

It is unknown if our results are biologically representative or a consequence of the experimental setup. Thus, generation of secretome in the high marrow cell density groups may have limited cell access to nutrients. This may have led to increased marrow cell stress or death, with resultant byproducts contributing to gene expression changes and reductions in MTT outcomes in the ST2 cell‐secretome co‐culture experiments. For example, Fig. 9 shows that when ST2 cells were cultured in high‐dose secretomes previously shown to reduce MTT outcomes, PDK4 gene expression was elevated and β actin gene expression was reduced. However, during secretome generation, the culture medium remained pink and never turned orange or yellow, which occurs with high‐density culture with high metabolic activity. Future experiments will include co‐culture in secretomes that did not impact MTT outcomes, as well as analysis of glucose and lactate levels in the secretomes themselves.

Relationships between secretomes generated in vitro and the in vivo bone marrow microenvironment should be taken into consideration. For example, lipids in the marrow can affect bone and marrow metabolism,^(^ ^8^, ^10^ ^)^ and lipid composition of secretomes compared with whole bone marrow should be addressed. Fatty acids were examined in primary bone marrow collected from a similar cohort of pediatric patients,^(^ ^19^ ^)^ but whether similar lipid profiles hold in cell culture models of the bone marrow microenvironment is an important consideration for future inquiry. In addition, mesenchymal stromal cells adhere more readily to tissue culture plastic compared with RBC and other hematopoietic cells and so secretomes generated over longer culture intervals may be enriched in MSC compared with the in vivo bone marrow milieu.

We also tested the contribution of red blood cells to the biological potency of marrow‐derived secretomes. RBC reduction attenuated the secretome‐induced reduction in ST2 cell MTT outcomes when secretomes were generated for 3 or 7 days using the highest cell dose (Fig. 3*B*, *C*). We were not able to completely eliminate RBC from the bone marrow samples. Similarly, RBC shed vesicles from their membranes^(^ ^26^ ^)^ that would not have been removed by chemical RBC lysis. Thus, at this time, we cannot completely rule out contributions of RBC‐associated factors to reduced MTT outcomes in ST2 cells cultured in marrow‐derived secretomes.

MTT is reduced to formazan by succinate dehydrogenase, which is a component of the electron transport chain (ETC) and the tricarboxylic acid (TCA) cycle. MTT can also be reduced by NADH and NADPH in mitochondria and cytosol. Formazan is membrane impermeable, so it accumulates in healthy cells. Thus, MTT outcomes are generally reflective of cell number. However, in this study, secretome formulations that had elicited significant reductions in ST2 cell MTT outcomes did not affect cell number or viability in subsequent cell culture experiments. Instead, these secretomes elicited a modest increase in PDK4 expression at 72 hours and a transient decrease in β‐actin expression at 24 hours.

PDK4 phosphorylates and inhibits pyruvate dehydrogenase (PDH), which converts pyruvate metabolized from glucose at the mitochondrial membrane into acetyl coA, which feeds into the TCA cycle.^(^ ^27^ ^)^ Increased PDK4 expression and inhibition of PDH activity correlate with a transition away from oxidative glucose metabolism to fatty acid oxidation.^(^ ^28^, ^29^ ^)^ Physical interactions between the actin cytosketon and mitochondria are known to regulate both cytoskeletal and mitochondrial dynamics.^(^ ^30^, ^31^ ^)^ The physiologic relevance of secretome‐induced changes in gene expression are not known, but differences in cell metabolism, rather than proliferation or survival, may explain our results.

We also measured expression levels of the transcriptional co‐activator ppargc1α because it regulates genes involved in the regulation of mitochondrial biogenesis^(^ ^32^ ^)^ and energy metabolism, including PDK4.^(^ ^33^ ^)^ However, our rtPCR results suggest that basal expression of ppargc1α in ST2 cells was very low, making detection of secretome‐dependent inhibition of expression difficult. Secretomes did not elicit increases in expression of this gene.

This study has limitations. Our sample size was small and precluded group comparisons. Healthy children and adolescents rarely undergo spinal surgery, and so collection of vertebral bone marrow from a true control group is challenging given the need to obtain informed consent from such a small number of potential participants. Thus our recruitment was restricted to convenience sampling from a small population of skeletally mature and immature patients who were undergoing spinal fusion surgery for neuromuscular scoliosis associated with CP of varying severity and Gross Motor Function Classification System (GMFCS) score and for idiopathic scoliosis. Further, bone marrow was only collected from the vertebrae, which may not fully represent the biology of bone marrow across other skeletal sites.^(^ ^34^, ^35^ ^)^ We did not characterize the cellular composition of the bone marrow samples or the chemical composition of the generated secretomes. The presence of serum during secretome generation may have influenced profiles of secreted factors or subsequent effects during ST2 cell co‐culture. Acquiring pediatric bone marrow is rare for research, and it allows for mechanistic investigations with direct clinical relevance. Comparing cellular, lipid, protein, and metabolomic constituents of primary whole bone marrow to the cells and secretomes present in cell models of the bone marrow milieu is an important consideration for future studies. As the goal of this study was to test how varying experimental conditions impacted generation of biologically active secretome in vitro, we did not examine secretome effects on post‐differentiation biological activities such as osteogenic or adipogenic differentiation capacity of mesenchymal stem cells. Here, we utilized the well‐studied ST2 cell line to standardize the phenotype of the cells exposed to the bone marrow secretomes. It is unknown if ST2 cell responses to secretome reflect responses of MSC to the bone marrow microenvironment in vivo, as there is little existing knowledge about intrinsic MSC traits from children.

In conclusion, pediatric bone marrow secretome collected during routine orthopedic surgery is biologically active in vitro. As outlined in Fig. 10, the findings from this study can inform design of future studies aimed at elucidating contributions of cell‐autonomous and non‐cell‐autonomous factors in the bone marrow to MSC differentiation potential, bone formation, and skeletal growth.

## Author Contributions

**Sanjana Kannikeswaran:** Investigation; writing – original draft; writing – review and editing. **Daniel G Whitney:** Conceptualization; funding acquisition; methodology; writing – review and editing. **Maureen J Devlin:** Writing – review and editing. **Ying Li:** Resources; writing – review and editing. **Michelle S Caird:** Resources; writing – review and editing. **Andrea I Alford:** Conceptualization; formal analysis; writing – review and editing.

## Conflicts of Interest

The authors declare no conflicts of interest.

### Peer Review

The peer review history for this article is available at https://www.webofscience.com/api/gateway/wos/peer‐review/10.1002/jbm4.10740.
